# Comparative genomics reveals a functional thyroid-specific element in the far upstream region of the *PAX8 *gene

**DOI:** 10.1186/1471-2164-11-306

**Published:** 2010-05-14

**Authors:** Roberto Nitsch, Valeria Di Dato, Alessandra di Gennaro, Tiziana de Cristofaro, Serena Abbondante, Mario De Felice, Mariastella Zannini, Roberto Di Lauro

**Affiliations:** 1 Institute of Experimental Endocrinology and Oncology 'G. Salvatore', National Research Council, via S. Pansini 5, Naples 80131, Italy; 2 Department of Cellular and Molecular Biology and Pathology, University of Naples Federico II, via S. Pansini 5, Naples 80131, Italy; 3 IRGS-Biogem, Ariano Irpino (AV), Italy

## Abstract

**Background:**

The molecular mechanisms leading to a fully differentiated thyrocite are still object of intense study even if it is well known that thyroglobulin, thyroperoxidase, NIS and TSHr are the marker genes of thyroid differentiation. It is also well known that Pax8, TTF-1, Foxe1 and Hhex are the thyroid-enriched transcription factors responsible for the expression of the above genes, thus are responsible for the differentiated thyroid phenotype. In particular, the role of Pax8 in the fully developed thyroid gland was studied in depth and it was established that it plays a key role in thyroid development and differentiation. However, to date the bases for the thyroid-enriched expression of this transcription factor have not been unraveled yet. Here, we report the identification and characterization of a functional thyroid-specific enhancer element located far upstream of the *Pax8 *gene.

**Results:**

We hypothesized that regulatory cis-acting elements are conserved among mammalian genes. Comparison of a genomic region extending for about 100 kb at the 5'-flanking region of the mouse and human *Pax8 *gene revealed several conserved regions that were tested for enhancer activity in thyroid and non-thyroid cells. Using this approach we identified one putative thyroid-specific regulatory element located 84.6 kb upstream of the *Pax8 *transcription start site. The *in silico *data were verified by promoter-reporter assays in thyroid and non-thyroid cells. Interestingly, the identified far upstream element manifested a very high transcriptional activity in the thyroid cell line PC Cl3, but showed no activity in HeLa cells. In addition, the data here reported indicate that the thyroid-enriched transcription factor TTF-1 is able to bind *in vitro *and *in vivo *the Pax8 far upstream element, and is capable to activate transcription from it.

**Conclusions:**

Results of this study reveal the presence of a thyroid-specific regulatory element in the 5' upstream region of the *Pax8 *gene. The identification of this regulatory element represents the first step in the investigation of upstream regulatory mechanisms that control *Pax8 *transcription during thyroid differentiation and are relevant to further studies on *Pax8 *as a candidate gene for thyroid dysgenesis.

## Background

The thyroid gland is a very important organ for the development of vertebrates as it synthesizes hormones that are essential for growth, development and survival such as tetraiodothyronine (thyroxine or T4) and triodothyronine (T3). Thyroglobulin (Tg), thyroperoxidase (TPO), sodium/iodide symporter (NIS), and TSH receptor (TSHr) are genes necessary for the synthesis of such hormones which takes place in the fully differentiated thyroid cell, called the thyrocite [1,2]. Indeed, some of these genes mark a differentiated thyroid cell; in particular, thyroglobulin and thyroperoxidase are genes exclusively expressed in thyroid cells. The promoters of these two genes have been extensively studied and three transcription factors, namely TTF-1 (also named Titf1/Nkx2-1), Foxe1 and Pax8, have been demonstrated to be involved in the activation of these genes [3,4]. During development and in the adult life, these factors are also present in other tissues, but the three of them are co-expressed only in the thyroid. It has been shown that their expression is required for the early stages of thyroid morphogenesis and is crucial for normal thyroid function. Indeed, for all its life a thyroid cell will be hallmarked by the simultaneous presence of TTF-1, Foxe1 and Pax8. Interestingly, these thyroid-enriched transcription factors are likely linked in a regulatory network such that each of them can be involved in the initiation or maintenance of the others [5].

During the past years, the role of Pax8 in the fully developed thyroid gland was studied in depth and it was established that Pax8 plays a key role in thyroid development and differentiation [6]. The first evidence of a role for Pax8 in the fully developed thyroid gland was provided by Mansouri et al. [7] by the generation of a Pax8 knockout mouse. Interestingly, Pax8^+/- ^mice had no phenotype, while homozygous Pax8^-/- ^mice showed growth retardation and died within 2-3 weeks. The cause of the death of the mutated animals was hypothyroidism, and the administration of thyroxine to Pax8^-/- ^mice allowed the animals to survive. In fact, these mice did not display any apparent defects in Pax8 territories of expression except for the thyroid gland that appeared smaller and no follicles were detectable, demonstrating that Pax8 is necessary for the survival of follicular thyroid cells. Furthermore, it was shown that in the thyroid anlage of Pax8^-/- ^mice the expression of Foxe1 is strongly down-regulated [5]. These observations demonstrated that Pax8 not only is required for the survival of thyroid precursor cells, but also holds a specific upper role in the genetic regulatory cascade, which controls thyroid development and its functional differentiation.

Indeed, the reintroduction *in vitro *of an exogenous Pax8 in the PCPy transformed thyroid cell line, in which Pax8 is absent as well as all the differentiation markers, was sufficient to re-activate transcription of the endogenous genes encoding thyroglobulin, thyroperoxidase and sodium/iodide symporter [8]. Furthermore, it has been reported that Pax8, together and synergistically with another thyroid-enriched transcription factor TTF-1, is able to activate transcription from the thyroglobulin gene promoter [9].

Although the function as well as the downstream targets of Pax8 are well studied, very little is known about its transcriptional regulation. Actually, an exhaustive knowledge of such a regulation is essential for a comprehensive view of thyroid gland development and differentiation pathways. Understanding those pathways is important not only to develop new treatments for thyroid gland diseases but also to add new dowels in the understanding of gene deregulation-dependent syndromes.

Therefore, the aim of this work was to shed light on the transcriptional regulation of Pax8 gene expression. The identification of distant acting gene regulatory sequences that direct precise spatial and temporal patterns of expression has been limited, despite their established roles in development, phenotypic diversity and human disease. Comparative genomic-based approaches have proved to be useful in identifying gene regulatory sequences, primarily on a gene-by-gene basis. These studies involved sequence comparisons of human (or other vertebrate) genomic intervals to orthologous regions from organisms separated by varying evolutionary distances, ranging from primates to fish [10].

We decided to use the bioinformatics approach to search for putative enhancer elements located in the 5'-flanking region of Pax8. We utilized a common interspecies sequence comparison and we analyzed 275 kb of orthologous regions of the human and mouse Pax8 gene. The rationale for using cross-species sequence comparisons to identify biologically active regions of a genome is based on the observation that sequences with important functions are frequently conserved among phylogenetically distant species, distinguishing them from non-functional surrounding sequences. We searched for non-coding genomic sequences (CNS), conserved between human and mouse, located in the 5'-flanking region of the Pax8 chromosomic locus, and we identified one sequence with a high thyroid-specific transcriptional activity. We demonstrated that this sequence is the first discovered enhancer of the Pax8 gene.

## Results

### Genomic comparison and identification of conserved non-coding sequences

**Figure 1:**
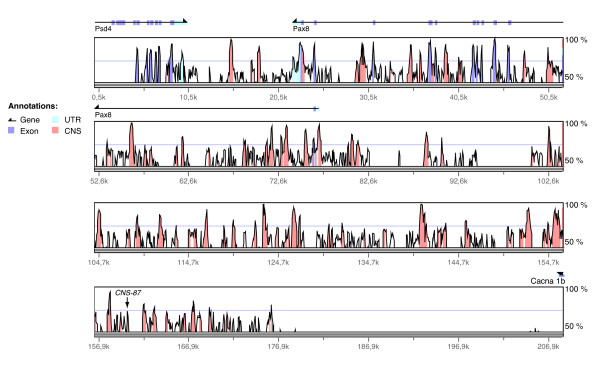
**Comparison of the Pax8 upstream and downstream genomic region between human and mouse**. Graphic output of the VISTA analysis of the mouse/human Pax8 genomic locus global alignment (for a better resolution 208 kb out of 275 kb analyzed are shown). On the x axis is represented the mouse sequence; the y axis indicates the percentage of similarity with the corresponding sequence in the human genome. The genes embedded in this part of the genome with their exons (violet), UTRs (pale blue) and direction of transcription are indicated. The identified CNS are shown as pink peaks.

Using the program VISTA, we compared 275 kb of orthologous regions of the human and mouse Pax8 gene searching for conserved non-coding sequences (CNS). The first known gene at the 3'-end and the first known gene at 5'-end of the Pax8 gene were taken as border limits of the genomic fragment selected. We considered sequences as conserved if they aligned without a gap for ≥100 bp and have ≥70% nucleotide identity. In Figure 1, the percentages of identical nucleotides are visualized as a peak along the sequence of the base organism (mouse; x axis). All peaks that cover pieces of sequences sharing more than 50% of similarity are drawn, and those corresponding to the settings described above are highlighted in pink. As confirmation, we used in parallel to the global comparison program VISTA, the local comparison program PIPMaker. The results of these two programs were completely overlapping, retrieving the same conserved sequences (data not shown).

91 CNS fitting the above-cited criteria were identified. We discarded 11 of them because of their overlap with coding sequences inside the Pax8 gene (exons and UTR) and the remaining 80 were defined as non-coding conserved elements. We decided to focus our attention on 32 segments in the 5'-flanking region of the Pax8 gene extending for 116 kb upstream the start of transcription and located more than 30 kb away from any known gene.

**Figure 2:**
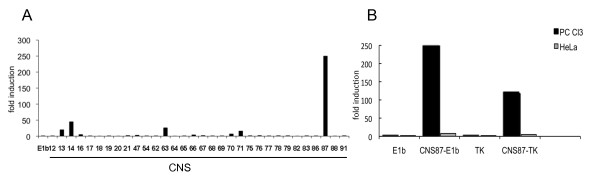
**Enhancer activity of the conserved sequences in the 5'-flanking region of the Pax8 gene**. A) All the CNS were subcloned upstream the minimal promoter E1b into the E1b-pGL3basic vector and transiently transfected in the rat thyroid cell line PC Cl3 (note: the CNS are not numbered in consecutive order). At 48 h post-transfection, transcriptional activity was determined as the firefly over renilla luciferase activity. Data are expressed as fold induction over the transcription obtained with E1b-pGL3basic, whose value was set at 1.0. Data are means from 3-4 independent experiments, each performed in duplicate; B) CNS87 was subcloned into the TK-pGL3basic vector (CNS87-TK) and its activity was compared with that of the CNS87-E1b vector in two different cell types. At 48 h post-transfection, transcriptional activity was determined as the firefly over renilla luciferase activity. Data are expressed as fold induction over the transcription obtained with the E1b-pGL3basic or the TK-pGL3basic vectors. Data are means from 3-4 independent experiments, each performed in duplicate.

To investigate the potential biological activity of these conserved regions, promoter-reporter assays were performed. To this end, each CNS was amplified by PCR on mouse genomic DNA and chimeric constructs were generated inserting each fragment in the pGL3-basic reporter vector containing the E1b minimal promoter upstream of the firefly luciferase. We then analyzed the ability of the 32 selected CNS to act as enhancer in the immortalized thyroid cell line PC Cl3. Cells were transiently transfected with the CNS-constructs together with an internal control plasmid (Renilla luciferase) to normalize for transfection efficiency. Figure 2A summarizes the transcriptional activity of each construct as fold induction over the transcription obtained with the E1b-pGL3-basic vector. First we noticed that, out of 32 CNS tested, only 7 showed transcriptional activity. Among these, the CNS14 and CNS87 showed the highest activity, with 45 and 250 fold increase respectively, over the basal level of the empty vector containing only the E1b basal promoter. Based on its position, coincident with the start of transcription, we hypothesized that the CNS14 could be a putative promoter and its analysis was conducted separately. For the continuation of the study we decided to characterize in better depth only the most active, 343 bp long CNS87, located 84.6 kb upstream of the Pax8 5'UTR (Figure 1).

Interestingly, the CNS87 manifested a very high transcriptional activity in the thyroid cell line PC Cl3, but it showed no activity in HeLa cells (Figure 2B). Similar results were obtained if the CNS87 was inserted in a reporter vector containing the minimal promoter of the thymidine-kinase gene (TK-pGL3basic). We conclude that the CNS87 is an enhancer with thyroid-specific transcriptional activity.

### The thyroid-specific transcription factor TTF-1 binds to the CNS87

**Figure 3:**
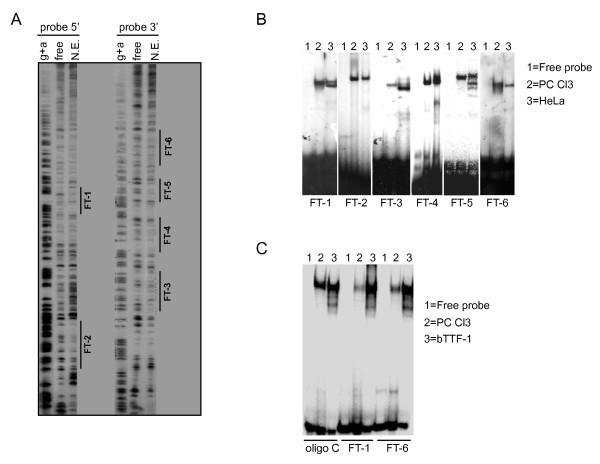
**DNAse I digestion of the CNS87 and EMSA assays**. A) DNAse I footprinting analysis of the CNS87 in the absence (-) or presence (+) of PC Cl3 protein extract. A sequence ladder of the CNS87 fragment (G+A) was used as size marker. The regions protected are indicated by lines and named FT-1 to FT-6; B) EMSA assays were performed to determine the cell-type specificity of the proteins binding to the CNS87. Protein extract from PC Cl3 (thyroid) and HeLa (non-thyroid) cells were used with a set of probes derived from the FT sequences; C) Bacterially-purified TTF-1 (bTTF1) was used in EMSA assays in comparison with PC Cl3 total protein extract. Oligo C was used as control. The retarded pattern is maintained in EMSA assays with FT-1 and FT-6 probes demonstrating that TTF-1 is binding to both probes.

We performed Dnase-I footprinting analysis using nuclear extracts prepared from PC Cl3 with ^32^P-5'end-labeled CNS87 and we observed six protected regions (here denominated FT) (Figure 3A). To further characterize proteins binding on each FT, we performed electromobility shift assays (EMSA) using 25 nt long oligonucleotides designed on each of the six FTs that were incubated with either thyroid (PC Cl3) or non-thyroid (HeLa) protein extracts (Figure 3B). While oligonucleotides derived from FT-2 and FT-4 showed similar retarded bands with both extracts, we noticed that the retarded bands corresponding to FT-1, FT-3, FT-5 and FT-6 oligo probes displayed completely different patterns when challenged with the two extracts. These results prompted us to investigate the possibility that on the CNS87 were present consensus sequences for thyroid-specific transcription factors. We then used the TRANSFAC analysis software (TRANSFAC pro^®^9.3 from the BIOBASE Company) to search for transcription factors binding sites and intriguingly, we found that FT-1 and FT-6 were predicted to have binding sites for the thyroid-enriched transcription factor TTF-1. Consequently, the remaining of our study was focused on these two footprints.

**Figure 4:**
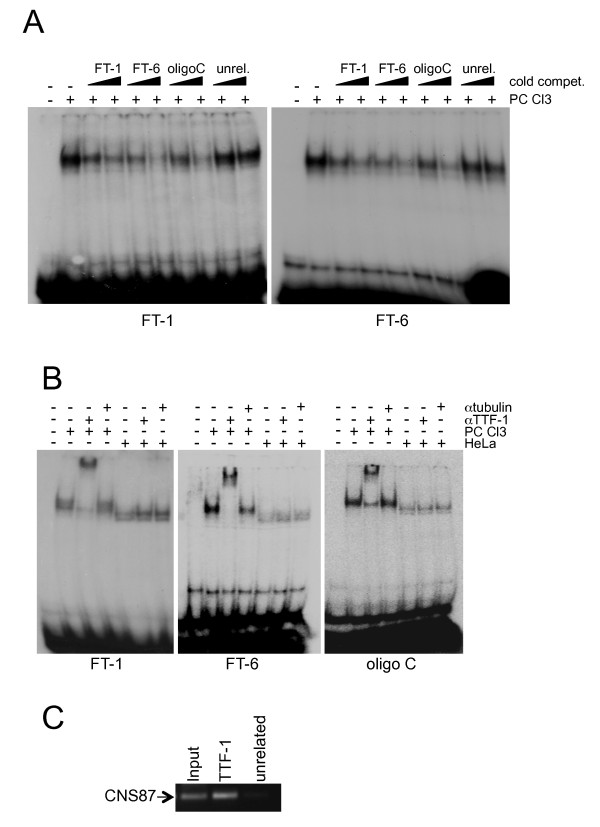
**TTF-1 binds to the FT-1 and FT-6 sequences**. A) Results from competition assays of the FT-1 and FT-6 retarded bands with increasing excess of the indicated cold competitors; B) Protein extract of PC Cl3 and HeLa cells were incubated, alone or together with the antibody against TTF-1 or tubulin (as control), in a binding assay with probes FT-1, FT-6 and oligo C. The PC Cl3 specific supershift observed with the antibody against TTF-1 is clearly visible; C) ChIP experiment performed on PC Cl3 cells showing the binding *in vivo *of TTF-1 to the CNS87 element.

To further characterize and verify the predictions obtained with the TRANSFAC analysis on FT-1 and FT-6, we proceeded with more exhaustive EMSA assays. Initially, we incubated the same FT-1 and FT-6 ^32^P-labelled oligo probes both with PC Cl3 total protein extract or with bacterially produced and purified TTF-1 protein (bTTF1) (Figure 3C). As control of the binding, we used the ^32^P-labelled oligo C described before [11], which is proved to bind the TTF-1 protein. As expected, bTTF1 binds to the oligo C and produces a retarded band similar to that observed with the total protein extract. Interestingly, the incubation of bTTF-1 with the FT-1 and the FT-6 oligos produced a retarded band identical to the one observed in the binding of bTTF-1 with the oligo C, suggesting that TTF-1 is indeed the protein binding to the FT-1 and FT-6 probes. In addition, the retarded band observed when the PC Cl3 protein extract was incubated with the FT-1 and FT-6 oligo probes is the result of a specific interaction, as demonstrated by competition assays (Figure 4A). Cross-competition further strengthened the data that indeed the FT-1, FT-6 and oligo C sequences bind the same protein (Figure 4A). To further consolidate our data, we performed supershift assays with an anti-TTF1 specific antibody. Also in this case, as shown in Figure 4B, we observed that to the supershifted band produced after the incubation of the oligo C with PC Cl3 protein extract and the anti-TTF1 antibody, exactly corresponds an identical supershifted band in the lanes corresponding to the FT-1 and FT-6 oligo probes. The specificity of these three supershifted bands is confirmed by the reduction of the expected retarded bands corresponding to TTF-1-oligo complex and by the absence of a supershift when the HeLa protein extract or the anti-tubulin antibody is used.

To demonstrate the ability of TTF-1 to interact with the CNS87 also *in vivo*, we performed chromatin immunoprecipitation (ChIP) experiments on PC Cl3 cells. The crosslinked chromatin was immunoprecipitated using the antibody against TTF-1. As control, to rule out unspecific background of the ChIP assay, we performed one reaction using an unrelated antibody. The enrichment of the endogenous CNS87 region was monitored by PCR amplification using specific primers. Indeed, we demonstrate that, in agreement with the *in vitro *data, TTF-1 antibody is able to immunoprecipitate the chromatin containing the CNS87 element (Figure 4C). This result confirms the *in vitro *binding data presented in this paper, and clearly demonstrate that the thyroid-enriched transcription factor TTF-1 is able to bind *in vivo *the Pax8 far upstream element CNS87.

### FT1 and FT6 are key regions for the transcriptional activity of the CNS87

**Figure 5:**
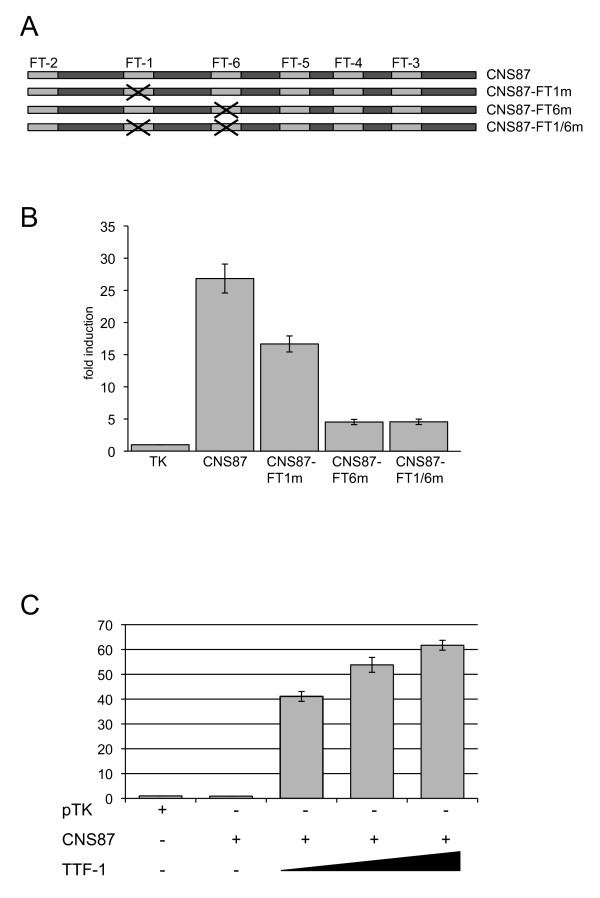
**Functional analysis CNS87 mutants**. A) Schematic representation of the footprints position on the CNS87 sequence. Three different CNS87 mutants were generated and cloned into the TK-pGL3basic vector as described for the wild-type sequence; B) All the CNS87 mutants were transiently transfected in PC Cl3 thyroid cells. At 48 h post-transfection, transcriptional activity was determined as the firefly over renilla luciferase activity. Data are expressed as fold induction over the transcription obtained with TK-pGL3basic, whose value was set at 1.0. Data are means ± SD from 3-4 independent experiments, each performed in duplicate; C) HeLa cells were transiently transfected with the CNS87-TK reporter construct in the absence or in the presence of increasing concentration of the expression vector encoding TTF-1. Folds of activation are considered as ratio between values obtained with and without co-transfection of the expression vector and the transcriptional activity was determined as the firefly over renilla luciferase activity. Data are means ± SD from 3-4 independent experiments, each performed in duplicate.

To better understand the functional role of the FT-1 and FT-6 regions in the transcriptional activity of the CNS87, we decided to mutagenize them by generating site-specific point mutations in both TTF-1 binding sites, separately or in combination. We chose the mutations with the help of the bioinformatics predictions of the TTF-1 binding consensus sequence such that on the CNS87 element TTF-1 binding was totally abrogated in the FT-1 and FT-6 regions and no other TTF-1 binding site was generated within the entire element. In this way, we prepared three new CNS87-TK vectors that were named CNS87-FT1m, CNS87-FT6m and CNS87-FT1-6m (Figure 5A). We used each of these three vectors in transient transfection experiments in PC Cl3 cells as described before, and we compared the results obtained transfecting these mutated vectors with the wild-type CNS87-TK. In Figure 5B, we show that the transcriptional activity of the CNS87-WT transfected in PC Cl3 cells is significantly higher then the transcriptional activities corresponding to each of the three mutants, indicating that both FT-1 and FT-6 are involved in the transcriptional activity of the CNS87. In particular, mutation in FT-6 gives the highest level of reduction in the transcriptional activity while the combination of mutations in both FT1/6 does not potentiate this reduction. These results led us to recognize as most critical the role of FT-6 among the other footprints present on the CNS87.

### TTF-1 activates transcription from the CNS87 and is involved in Pax8 expression in thyroid cells

Since TTF-1 was the only confirmed binding factor on the CNS87, and to better understand its role in the transcriptional activity of this element, we decided to perform transactivation experiments in a heterologous recipient. Hence, we transfected HeLa cells with the wild-type CNS87 vector alone or in co-transfection with an expression vector encoding TTF-1. The results showed in Figure 5C demonstrated that the co-transfection of the transcription factor TTF-1 is capable to strongly activate transcription from the CNS87, and in particular the fold activity of luciferase expression obtained in co-transfection experiments clearly resembles the levels obtained transfecting the CNS87 in the physiological PC Cl3 cell recipient.

**Figure 6:**
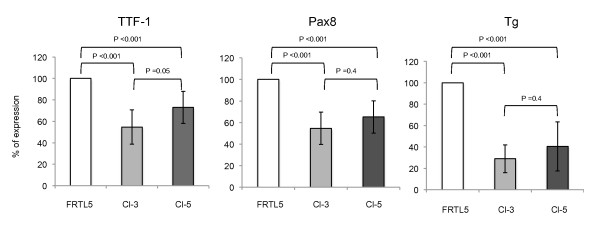
**TTF-1 plays a role in the regulation of Pax8 expression in differentiated thyroid cells**. Q-PCR analysis was performed on total RNA prepared from FRTL-5 rat thyroid cells and two clones, Cl-3 and Cl-5, stable transfected with an shRNA driven against TTF-1. The expression of TTF-1, Pax8 and Tg genes was measured. For each gene, values are means ± SD of seven independent experiments in duplicate, normalized by the expression of β-actin, and expressed as percentage of the value measured in parental FRTL-5 cells. Statistical analysis uses *t *test.

To better elucidate and confirm the role of TTF-1 in the regulation of Pax8 expression, we used the experimental model described by Dentice et al. [12] in which TTF-1 gene expression is stably knocked down. This cell model consists of a thyroid-specific cell line FRTL-5 stable transfected with an shRNA vector driven against TTF-1 mRNA. Among the selected clones, Cl-3 and Cl-5 showed a significantly reduced TTF-1 expression. We performed Q-PCR analysis on total RNA prepared from these two clones and from control untransfected FRTL-5 cells. We confirmed that in both clones TTF-1 expression was reduced and we showed that to the reduction of TTF-1 expression clearly corresponds a significant reduction of Pax8 expression (Figure 6), indicating that the level of expression of the two transcription factors is indeed correlated. To further demonstrate that the cell model used reflects the physiology of the thyroid cell, we analyzed by Q-PCR the expression in Cl-3 and Cl-5 also of one important thyroid differentiation marker, i.e. the thyroglobulin (Tg) gene that is a well known target of both TTF-1 and Pax8 [8,13]. As expected, Tg gene expression was significantly reduced in both Cl-3 and Cl-5 (Figure 6). The fact that Tg decreases more than the two transcription factors could be the consequence of the synergic action of TTF-1 and Pax8 on the Tg promoter, demonstrated both in vitro [9] and in vivo [14].

All together these data confirm that TTF-1 is responsible for the full expression of the Pax8 gene and fit perfectly with our previous data in which we demonstrated the transcriptional activity of TTF-1 on the Pax8 far upstream enhancer CNS87.

### CNS87 is a distant regulatory element that controls Pax8 expression

**Figure 7:**
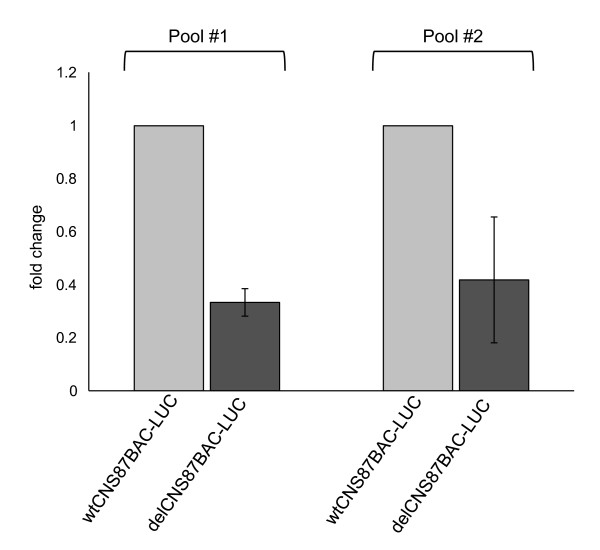
**A BAC-based Pax8 transcriptional reporter construct reveals the role of the CNS87 element in the control of Pax8 expression levels**. PC Cl3 cells were stable transfected with two reporter constructs derived from a BAC containing the entire Pax8 gene as well as the 5'-flanking region engineered as described in Materials and Methods. Pools of colonies for each construct were analyzed. Protein extracts were prepared and the luciferase activity was measured. Data are expressed as fold change with respect to the transcriptional activity obtained with wtCNS87BAC-LUC, whose value was set at 1.0. Data are means ± SD from 3-4 independent experiments, each performed in duplicate. Statistical analysis uses *t *test (p < 0.05).

To verify whether Pax8 expression could be regulated by a distant cis-acting sequence like the CNS87 element that we had identified, we used bacterial artificial chromosome (BAC) vectors. The Pax8 genomic locus is located on chromosome 2 with a length of about 60 kb. As previously described, the CNS87 element is located about 90 kb upstream the Pax8 5'UTR so both the Pax8 gene and the CNS87 sequence largely fit into a BAC vector. We obtained several BACs from the BAC PAC Resources of the Children's Hospital Oakland Research Institute (CHORI, http://bacpac.chori.org/) that respected the reported parameters and we looked for the presence of both Pax8 gene and the CNS87 into the same BAC vector with a PCR strategy (data not shown). Once we identified the BAC useful for our studies, we followed the convenient recombineering strategy to genetically engineer the selected BAC, in order to obtain that the BAC vector expresses the luciferase reporter gene instead of the Pax8 gene. This LUC-containing BAC vector, named wtCNS87BAC-LUC, can still be considered wild type with respect to the whole region upstream the Pax8 gene, that is the region containing the CNS87 and the putative promoter. To unravel the role of the CNS87 in its physiological genomic context, we further modified the wtCNS87BAC-LUC vector, again by means of recombineering, to eliminate the entire CNS87 region that was replaced by an ampicillin-resistance cassette (delCNS87BAC-LUC). We then used these two BAC vectors in stable transfections assays in PC Cl3 cells. Cells were transfected with wtCNS87BAC-LUC or with delCNS87BAC-LUC and after a 2-weeks G418 selection two different pools of stable clones were generated for each of the transfected vector. Protein extracts were prepared and the luciferase activity was measured. The results, reported in Figure 7, showed a strong transcriptional activity of the wtCNS87BAC-LUC as it was expected since, as mentioned above, Pax8 putative promoter and every other transcriptional element are preserved in this BAC vector. On the other hand, the level of luciferase expression corresponding to the delCNS87BAC-LUC vector is significantly reduced compared to the wtCNS87BAC-LUC luciferase expression. The absence of any other difference between the two BAC vectors, with the exception of the CNS87 deletion, led us to the conclusion that the CNS87 is a distant regulatory element that controls the expression level of the Pax8 gene.

## Discussion

Comparative analysis of non-coding sequences between evolutionary correlated species is one of the new tools of the genomic era widely used when researchers want to look for regulatory mechanisms that are conserved throughout evolution. Such an approach in fact leads to the identification of highly conserved sequences that may be functionally relevant and to the discovery of new transcription factors binding sites and mechanisms that were to be known yet. Inter-genomic comparisons indeed, are rapidly evolving for investigations of regulatory regions involved in promoter activity [15-19].

Pax8, a thyroid-enriched transcription factor, plays a key role in thyroid development and differentiation [8]. Despite its essential function, the transcriptional regulation of the Pax8 gene remains poorly characterized. In this paper, we describe the identification of the first transcriptional regulatory element of the PAX8/Pax8 gene. We achieved our results by coupling modern computational studies with experimental EMSA analysis and promoter/reporter assays.

We performed comparative studies on human and mouse genomic sequences flanking the Pax8 gene looking for conserved sequences to explore the possibility that such conserved non-coding sequences (CNS) may be instrumental in guiding the thyroid-specific expression of Pax8. By globally aligning a large fragment of the human and mouse Pax8 genomic locus, we found about 80 CNS that fitted our search criteria. However, we decided to focus our attention only on 32 of those sequences located in the 5'-flanking region of the Pax8 gene extending for about 110 kb upstream the start of transcription. Using promoter/reporter assays, we identified one conserved and functional sequence with a typical enhancer behavior specifically acting in thyroid cells and we named this sequence CNS87 (conserved non-coding sequence 87). In particular, this conserved sequence is a far upstream element, conserved between human and mouse, that turned out to be a target of the action of another thyroid-enriched transcription factor, i.e. TTF-1/NKX2.1.

Using several mutant mouse lines, Parlato et al. have established that in thyroid cell precursors the transcription factor TTF-1 and Pax8 are linked in a complex network of reciprocal regulatory interaction [5]. Our data strongly support this scenario indicating that TTF-1, through the CNS87 element, is involved in the regulation of Pax8 gene expression in the environment of the thyroid cell. In fact, the CNS87 has all the features typical of an enhancer since it can stimulates the transcription of a reporter gene in a very strong and tissue-specific manner. A peculiar feature of the CNS87 element is its location about 90 kb upstream from the 5'UTR of the mouse/human Pax8 gene. It is noteworthy that a sequence located so far from a gene could be related with its expression. However, many examples exist in the literature on regulatory elements located very far from both the 5'end and the 3'end or even inside other genes. The most representative example came from *Sonic Hedgehog *gene (Shh), where its regulatory element is located 1 Mb upstream of Shh, embedded in a gene that resides in a cluster of unrelated genes [20-23]. In addition, many model of action have been developed for long distance acting regulatory elements. Such models imply the existence of factors that have the ability to bring and stimulate genes in active compartment of the chromatin. Therefore regulatory elements get closer to the proximal regulatory region of a gene allowing the start of the transcription [24].

The notion that the CNS87 could indeed serve as a regulatory element of Pax8 gene expression was further reinforced by the experimental observation with the BAC transfection assays. Those data clearly confirm that the CNS87 fragment is required for the full expression of the Pax8 gene.

The bioinformatics predictions of the transcription factors binding sites in this CNS element further highlight the physical experimental data. The TRANSFAC program predicted two binding sites for the thyroid-enriched transcription factor TTF-1 within footprints 1 and 6. Indeed, when the sequences corresponding to the FT-1 and FT-6 are mutated the CNS87 activity is severely reduced respect to that of the wild-type fragment confirming that TTF-1 is leading the activity of this element.

## Conclusion

Taken together, our data suggest that the transcription factor TTF-1 participates to Pax8 gene expression directly binding to its 5'-flanking region and activating transcription from the CNS87 regulatory element that we have identified and characterized. In conclusion, this paper puts a milestone in the understanding of the regulation of the expression of the transcription factor Pax8 and will contribute to the establishment of a fine intergenic network between thyroid enriched transcription factors and their roles during development and adult life.

## Methods

### Sequence Analysis

Mouse and human DNA sequences were obtained from the University California Santa Cruz Genome Browser (mouse genome, release Feb 2007; human genome, release June 2007). From this database, a region was selected spanning 275 kb of the entire Pax8 genomic locus in Homo sapiens and Mus musculus. The first known gene at the 3' and the first known gene at 5' of the Pax8 gene were taken as border limits of the genomic fragment selected. The genomic fragment was first chosen from the mouse genome. Then, the corresponding genomic fragment from the human genome containing the same size of DNA at the 5' and 3' flanking sides of the PAX8 gene (77.653 bp at the 3' and 140.982 at the 5') was taken. Comparative genomic analysis was carried out using the PipMaker http://bio.cse.psu.edu/pipmaker 51 and VISTA http://www.gsd.lbl.gov/vista/ 50 programs. The conserved sequences identified in this way were analyzed and compared with the TRANSFAC 4.0 database using Mat Inspector vertebrate matrix database to search for potential transcription factor binding sites. Sum of both minimized false positive and false negative errors were considered to ensure proper interpretation of the survey results.

### PCR Amplification and subcloning of the Conserved Sequences

32 CNS positioned at the 5' of the Pax8 transcription start site or immediately downstream of it were selected for experimental test. These fragments were amplified by PCR using Platinum Taq DNA Polymerase High Fidelity (Invitrogen) and the PCR products were separated on a 2% agarose gel electrophoresis stained with ethidium bromide.

The conserved DNA sequences amplified by PCR were cloned in the pGL3basic vector (Promega) containing an E1b TATA box upstream the Luciferase reporter gene (pGL3-LUC), or into pGL3-TK vector containing the thymidine-kinase promoter [25].

The constructs CNS87-FT1m, CNS87-FT6m and CNS87-FT1-6m containing the mutated sequences of the CNS87 were generated by recombinant PCR.

### Preparation of Cell Nuclear Extract and DNaseI Footprinting Assay

Total cell extracts were prepared from PC Cl3 cells grown in one hundred plates (diameter, 150 mm) as previously described [26]. The footprinting probes were generated by digestion of the p87-E1b-LUC plasmid. The plasmid was linearized with the restriction enzyme XhoI, dephosphorylated with calf intestinal alkaline phosphatase (Roche Diagnostics) and extracted from agarose gel using the Qiaquick gel extraction Kit (Qiagen). 1.24 μg of linearized and purified plasmid DNA were end-labelled with γ-ATP-32P using T4 DNA polynucleotide kinase (BioLabs). The reverse strand probe was prepared in a similar way using as restriction enzyme Mlu1. The end-labelled probes were released from the plasmid with a second digestion and purified on a 5% acrylamide gel. 7 kcpm of the eluted probes were digested for 1 min at 20°C with 1:5000 and 1:2000 serial dilutions of a 3 mg/ml DNaseI (Roche Diagnostics) stock solution. The eluted probes were incubated with 30 μg of PC Cl3 nuclear extract and digested for 1 min at 20°C with 1:100, 1:50 and 1:25 serial dilutions of a 3 mg/ml DNaseI (Roche) stock solution.

### Cells and Transient Transfection Assay

PC Cl3 and FRTL-5 cells were grown in Coon's modified F-12 medium (Euroclone) supplemented with 5% v/v calf serum and a six-hormone mixture (6H), as described [27].

HeLa cells were grown in Dulbecco's modified Eagle's medium (Euroclone) supplemented with 10% v/v fetal calf serum (Hyclone). For transient transfection experiments, cells were plated at 3 × 10^5 ^cells/60-mm tissue culture dish 5 to 8 h prior to transfection, whereas PC Cl3 cells were plated at a density of 5 × 10^5 ^cells/60-mm tissue culture dish 18 h prior to transfection. Transfections were carried out with the FuGENE6 reagent (Roche Diagnostics) according to the manufacturer's directions. The DNA/FuGENE ratio was 1:2 in all the experiments. The plasmid pRL-TK (Promega) was used as internal control in the transfection assays. Cells extracts were prepared 48 h after transfection to determine the levels of the firefly and renilla luciferase with the Dual Luciferase Reporter Assay System (Promega). Transfection experiments were done in duplicate and repeated at least three times.

### Protein extracts and Gel Mobility Shift Assay

Cells were washed twice with ice-cold phosphate-buffered saline (PBS) and lysed in a buffer containing 10 mM Hepes pH 7.9, 400 mM NaCl, 0.1 mM EGTA pH 7.8, 5% v/v glycerol, 1 mM dithiothreitol (DTT), 1 mM phenylmethylsulfonyl fluoride (PMSF).

TTF-1 purified protein (bTTF1) was produced as previously described [28]. For EMSA assays, double-stranded oligonucleotides were labeled with γ-^32^P ATP and T4 polynucleotide kinase (New England BioLabs) and used as probes. The binding reactions were carried out in a buffer containing 10 mM HEPES (pH 7.9), 10% glycerol, 0.1 mM EDTA, 8 mM MgCl_2_, 1 mM dithiothreitol, 0.15 μg/ml of poly (dI-dC) for 30 min at room temperature. DNA-protein complexes were resolved on a 6% nondenaturing polyacrylamide gel and visualized by autoradiography.

The antibodies, αTTF-1 [29] and αtubulin (sc5286, Santa Cruz), used in the supershift experiments were incubated with the protein extract for 20 min before adding the probe.

Oligonucleotides were derived from the protected sequences in the footprinting assays on the CNS87 and were as follows: FT-1: CGCACAAGAGCCCTTCTCAAGGGAT; FT-2: CTGGCTAAAGCCCAACGACACAGGT; FT-3: AACACTTGGGTGATCTACGTGAAGC; FT-4: GGGGGCAGGTTGGACAAAAGCCCCA; FT-5: CCTCAACAGCTTCTGACCTTCCTCT; FT-6: GAGAACGTTTATAAGTGTCTGGCTG.

### RNA Extraction, cDNA Synthesis, and Real time-PCR

Total RNA was prepared using TRIZOL Reagent (Invitrogen) according to the manufacturer's directions. Total RNA (1 μg) was retrotranscribed using the iScript cDNA Synthesis kit (Bio-Rad). Real-time PCR analysis was performed using an iCycler-iQ real-time detection system and SYBR green chemistry (Bio-Rad, Hercules, CA). Reactions were carried out in duplicate in four independent experiments. The specific primers sets used for this analysis were for amplification of β-actin (Fwd: GGCAATGAGCGGTTCCGATG; Rev: ATGGTGGTGCCACCAGACAG), of Pax8 (Fwd: CAGCTATGCCTCTTCCGCTATT; Rev: TGTGGCTGTAGGCATTGCC), for TTF-1 (Fwd: AGGACACCATGCGGAACAGC; Rev: GGCCGCCCATGCCGCTCATA) and thyroglobulin (Fwd: TGTGGAATCTAATGCCAAGAACTG; Rev: TCCCTGAGAGCTTTTGGAATG).

For each gene, values are means ± SD of four independent experiments, normalized by the expression of β-actin, and expressed as a percentage of the value measured in parental FRTL-5 cells.

### Chromatin Immunoprecipitation

The cross-linking solution, containing 1% formaldehyde, was added directly to cell culture media. The fixation proceeded for 10 min and was stopped by the addition of glycine to a final concentration of 125 mM. PC Cl3 cells were rinsed twice with cold PBS plus 1 mM PMSF, and then scraped. Cells were collected by centrifugation at 800 × g for 5 min at 4 C. Cells were swelled in cold cell lysis buffer containing 5 mM piperazine-N, N'-bis(2-ethanesulfonic acid) (pH 8.0), 85 mM KCl, 0.5% Nonidet P-40, 1 mM PMSF, and inhibitors cocktail (Sigma) and incubated on ice for 10 min. Nuclei were spun down by microcentrifugation at 2000 × g for 5 min at 4 C, resuspended in nuclear lysis buffer containing 50 mM Tris-HCl (pH 8), 10 mM EDTA, 0.8% sodium dodecyl sulfate (SDS), 1 mM PMSF and inhibitors cocktail (Sigma), and then incubated on ice for 10 min. Samples were broken by sonication into chromatin fragments of an average length of 500/1000 bp and then microcentrifuged at 16,000 × g. The sonicated cell supernatant was diluted 8-fold in ChIP Dilution Buffer containing 0.01% SDS, 1.1% Triton X-100, 1.2 mM EDTA, 16.7 mM Tris-HCl (pH 8.1), and 167 mM NaCl, and precleared by adding Salmon Sperm DNA/Protein A Agarose (Upstate Biotechnology, Inc., Lake Placid, NY) for 30 min at 4 C. Precleared chromatin from 1 × 10^6 ^cells was incubated with 1 μg of affinity-purified rabbit polyclonal antibody, αTTF-1 [29] and an unrelated one, rotated at 4 C for 16 h. Immunoprecipitates were washed five times with RIPA buffer containing 10 mM Tris-HCl (pH 8), 1 mM EDTA, 1% Triton X-100, 0.1% Na-deoxycholate, 0.1% SDS, 140 mM NaCl, and 1 mM PMSF; twice with LiCl buffer containing 0.25 M LiCl, 1% Nonidet P-40, 1% Na-deoxycholate, 1 mM EDTA, 10 mM Tris-HCl (pH 8.0), and then three times with TE (10 mM Tris-HCl, pH 8; 1 mM EDTA). Before the first wash, the supernatant from the reaction lacking primary antibody was saved as total input of chromatin and was processed with the eluted immunoprecipitates beginning at the cross-link reversal step. Immunoprecipitates were eluted by adding 1% SDS, 0.1 M NaHCO_3 _and reverse cross-linked by addition of NaCl to a final concentration of 200 mM and by heating at 65 C for 16 h. Recovered material was treated with proteinase K, extracted with phenol-chloroform-isoamyl alcohol (25:24:1) and precipitated. The pellets were resuspended in 30 μl of TE and analyzed by PCR using specific primers for the CNS87. The input sample was resuspended in 30 μl of TE and diluted 1:10 before PCR.

### BAC

Mouse BAC clone bMQ-241M1 was obtained for the bMQ mouse BAC library made at the Wellcome Trust Sanger Institute constructed from the 129SvEv/AB2.2 mouse strain. Engineered versions were produced via recombinogenic targeting in Escherichia coli by the method of Lee et al. [30], also know as recombineering. Briefly, a luciferase expression cassette with a neo resistance cassette was inserted in the exon 2 of Pax8 into the ATG start codon. To obtain this construct luciferase cDNA form PGL3 was cloned upstream PGK-EM7-Neo cassette from pl452.

The recombination cassette was constructed by subcloning a 50-bp 5' recombination arm overlapping part of pax8 intron 1 and part of exon 2 upstream ATG start codon and a 50-bp 3'arm recombination arm containing part of exon 2 and intron 2 downstream ATG start codon such that the recombination arms flanked the Luc-PGK-EM7-Neo cassette. The forward strand sequences of the 50-bp homology arms were as follows, for the 5'arm: TGCGTAGGAAAGCTGCGAGTGTCCCTCAGTCTGTGAGCGACTCCCCGGCG; for the 3' arm: ATGCCTCACAACTCGATAGATCCGGTAAGGACCGCGGAGGGGCCAGGAC.

The final cassette with recombination arms was digested from the vector, gel purified and then recombined with pax8 BACs as described previously. Successful recombinants BAC Luc-Neo were selected by plating the electroporated cells on LB plates containing 20 μg/ml Kanamycin, 20 μg/ml chloroamphenicol. Correct modified BACs were verified by PCR analysis. For BAC 87- (lacking 87 region) the BAC luc-Neo was modified by insertion of Amp-resistance cassette into the 87 sequence. Amp-resistance cassette was amplified from pBS vector using primer 5' flanked by recombination arm overlapping the sequence upstream 87 region and using 3' primer flanked by recombination arm overlapping sequence downstream 87 region.

The forward strand sequences of the 50-bp Homology arms were as follows, for the 5'arm: AAAGAGAGGCAAAGAAAGCTAGGGGTCTGCAGTCTCCAAACCTGCAGGGCTGGCTAAAG; for the 3'arm: TTTCTGAATCTAAATCCAAAACTTTACCCTCTTCTGATTGGTAATGAGTC.

The PCR product was purified and recombined with BAC Luc-Neo. Recombinants were selected by plating the electroporated cells on LB plates containing 20 μg/ml Kanamycin, 20 μg/ml chloroamphenicol and 50 μg/ml ampicillin and the correct modified BACs were verified by PCR analysis.

### BAC transfection

BAC DNA for transfection was prepared with Qiagen Large-Construct kit.

Stable transfection were carried out with PEI22 (MBI Fermentas, St.Leon-Rot, Germany) as described previously [31]. PC Cl3 cells were seeded in 6-well dishes (2 × 105) the day before transfection. For each construct 1 μg of BAC DNA and 2 μg of BAC DNA were transfected with an amount of PEI giving an N/P ratio of 7.5. The medium was replaced on day 3 with fresh medium containing 150 μg/ml G418. Stably transfected cell pools were grown to confluence. Cells were then washed twice with phosphate-buffered saline and incubate for 30 min at room temperature with passive lysis buffer (Promega) with vigorous shaking. Firefly luciferase activity was determined using "Luciferase assay System" (Promega). Luciferase activity was then normalized with protein concentration of cleared lysates.

## Authors' contributions

RN carried out the biochemical assays and drafted the manuscript, VDD carried out the comparative genomic analysis and the biochemical assays, ADG performed the BAC experiments, SA carried out biochemical assays and molecular studies, TdC carried out molecular studies, MDF and MZ participated in the design of the study and analyzed the results, RDL was responsible for the coordination and supervision of the entire study. All authors read and approved the final manuscript.
